# Analysis of Survival in Complete Pathological Response after Long-Course Chemoradiotherapy in Patients with Advanced Rectal Cancer

**DOI:** 10.3390/curroncol30010081

**Published:** 2023-01-12

**Authors:** Cemal Ulusoy, Gülçin Harman Kamalı, Andrej Nikolovski

**Affiliations:** 1 Department of General Surgery, Prof. Dr. Cemil Taşçıoğlu Şehir Hastanesi, Istanbul 34384, Turkey; 2 Department of Pathology, Prof. Dr. Cemil Taşçıoğlu Şehir Hastanesi, Istanbul 34384, Turkey; 3 Department of Visceral Surgery, University Surgical Clinic “Sv. Naum Ohridski”, Ss. Cyril and Methodius University in Skopje, 1000 Skopje, North Macedonia

**Keywords:** complete pathological response, neoadjuvant chemoradiotherapy, rectal cancer, survival, tumor regression grade

## Abstract

Background: Neoadjuvant chemoradiotherapy prior to surgery is the standard treatment for locally advanced rectal cancer. This consists in the patient’s complete pathological response being achieved with no residual tumor presence in the resected specimen, which results in survival improvement. Methods: This retrospective study aimed to examine the rate of complete pathological response in patients with advanced rectal cancer treated with neoadjuvant long-course chemoradiotherapy and to examine the survival differences between the different tumor regression grade (TRG) scores. Results: A total of 154 patients were operated prior to long-course chemoradiotherapy with a total of 50 Gy plus FOLFOX protocol. Complete pathologic response was achieved in 29 (18.8%) patients. There was no statistical difference for the different pathologic responses according to gender, type of surgery, and number of harvested lymph nodes. Mean survival for all the groups was 37.2 months. Survival within a different TRG score exhibited statistical significance (*p* = 0.006). Overall, the survival rate during the follow-up period was of 81.8%. Conclusions: The complete pathological response rate in this study was of 18.8%. High tumor regression grade scores (TRG0 and TRG1) had a survival rate of over 90% during follow-up. Multivariate analysis identified perineural invasion and tumor regression grade as independent factors that affect survival.

## 1. Introduction

Rectal cancer is the third most predominant malignancy that affects the population with the current highest incidence in Australia and it is strongly related with age. A sharp increase in its occurrence is noted after the age of 50. A slight increase in the rectal cancer incidence has been reported in younger age (below 50) in the last decade [1]. Rectal cancer treatment is still undergoing major changes in terms of the proper modality treatment choice [2]. After the establishment of a combination of neoadjuvant treatment with total mesorectal excision (TME) [3] for advanced rectal cancer, changes followed in recent years. The proposed specter of different treatment modalities ranges from combined neoadjuvant short-course radiation, through long-course neoadjuvant chemoradiotherapy followed by surgery to “watch and wait” strategy and the total neoadjuvant treatment [4,5]. As a result, the type of surgery for rectal cancer changed in favor of procedures that spare the sphincter apparatus, while part of the patients are never operated due to the advanced treatment modalities [6,7].

Although the “watch and wait” strategy became part of the clinical practice guidelines [7], the combination of neoadjuvant chemoradiotherapy (CRT) and surgery remains the standard current treatment for stage II/III rectal cancer. By adding the neoadjuvant regiment, tumor downsizing and local nodal downstaging is reported in some patients. It provides high rates of negative resection margin achievement and sphincter preservation at the same time [8]. As a result, with the improvement of the overall survival, disease-free survival and local recurrence rates decrease became the reality [9,10,11].

The use of neoadjuvant chemoradiation can lead to a certain degree of tumor regression, and the same can be confirmed only after radical rectal cancer surgery. Different tumor regression scores have been used in order to measure the tumor regression grade (TRG) [12,13,14,15]. The reported rate of complete pathological response (cPR) is between 10 and 30% [3,16,17]. Patients with cPR achievement are reported to have excellent disease-free and overall survival rates [18,19].

Still, in the vast majority of patients, the pathological response is incomplete or poor, thus leading to undesirable effects after the neoadjuvant chemotherapy. In addition to this, controversial data are reported on whether cPR leads to improvement in survival [20,21,22]. Further analysis of the effect of different TRGs on survival has been proposed [23]. This single-center study focuses on the degree of cPR achievement after long-term neoadjuvant chemoradiation in patients with stage II/III rectal cancer; it estimates the survival differences between different grades of tumor regression and identifies factors that affect survival.

## 2. Materials and Methods

This retrospective study analyzes the grade of pathological response after neoadjuvant CRT and the survival within the different grades of pathological response in patients with stage II and III rectal cancer. In the period of January 2016–January 2022, patients with diagnosed rectal cancer were operated upon following the decision on neoadjuvant treatment conduction. The oncologic board decision was based on the preoperative clinical stage of the tumor. Long-course chemoradiotherapy was conducted with a total of 50 Gy divided into 25 fractions plus FOLFOX (5-FU, oxaliplatin, and leucovorin). Re-evaluation after neoadjuvant CRT was mandatory and encompassed magnetic resonance imaging of the pelvis and endoscopic examination (performed by the attending surgeon). Two curative procedures (low anterior resection with total mesorectal excision and extralevator/abdominoperineal rectal excision) were employed. Regardless of the procedure, high ligation of the inferior mesenteric artery was always performed considering the oncologic principles. The timing for surgery after neoadjuvant treatment completion was 8–12 weeks. Patients were referred to oncologists after hospital discharge. One year after surgery, routine follow-up colonoscopy was scheduled. Patient characteristics, surgery and tumor data, and follow-up period were collected. Postoperative pathological evaluation of the resected specimens was conducted by 3 different pathologists.

Tumor regression grade on the pathological response grade was performed by the use of The American Joint Committee of Cancer and College of American Pathologists (AJCC/CAP) tumor regression grading (TRG) four-category system. The categories of the system comprehend the following grades: TRG0 (complete response with no viable cancer cells in the specimen), TRG1 (moderate response with present small cluster or single cancer cell remaining), TRG2 (minimal response with residual cancer and predominant fibrosis), and TRG3 (poor response with minimal or no tumor kill with extensive residual cancer) [15]. According to TRG, patients were divided into four groups. Survival analysis differences were performed between the 4 groups with different TRGs.

The study was approved by the local ethical committee of the hospital, Protocol No. E-48670771-514.99, decision No. 344. IBM SPSS, version 25 (IBM Corp., Armonk, NY, USA), which was used for statistical analysis. Variable distribution normality was tested with Kolomogorov–Smirnof test. Chi-squared test was used for two categorical variables comparison. Analysis of variance (ANOVA) test was used to compare survival between different TRG groups. Kaplan–Meier method and the log rank test were used to compare differences between the groups in terms of overall survival. Multiple Cox proportional hazard regression analysis was performed to identify the factors that affect survival. *p* value of less than 0.05 was considered statistically significant.

## 3. Results

A total of 154 patients were operated with either low anterior resection of the rectum (105), with extralevator abdominoperineal excision of the rectum (24), or with conventional abdominoperineal rectal resection (25). According to gender distribution, 105 were male and 49 were female patients. The mean age of the patients was 61.1 years. Stage II was present in 65 patients and the remaining 89 presented with stage III. Metastases in local lymph nodes were detected in 56 (36.4%) patients. Lymphovascular invasion (LVI) and perineural invasion (PNI) presence were detected in 30 (19.5%) and 47 (30.5%) patients, respectively. There was no statistically significant difference between gender according to tumor stage, LVI, and PNI presence (Table 1).

**Figure 1:**
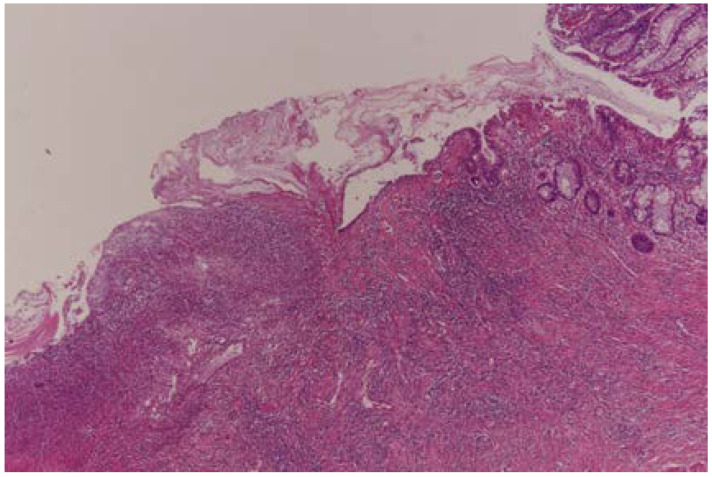
Complete pathologic response (TRG0); x 40 HE; Mucosal ulcer area, no residual tumor.

**Figure 2:**
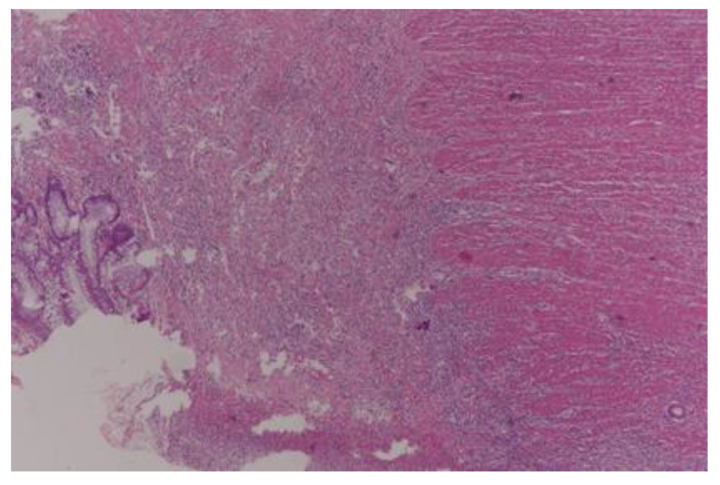
Moderate pathological response (TRG1); x 40 HE; Mucosal ulcer, glandular tumor in muscularis propria.

**Figure 3:**
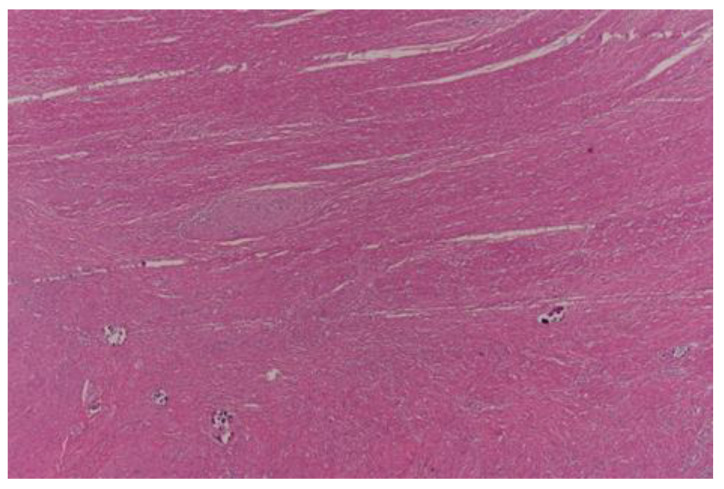
Moderate pathological response (TRG1); x 40 HE; Calcifications in the muscularis propria.

**Figure 4:**
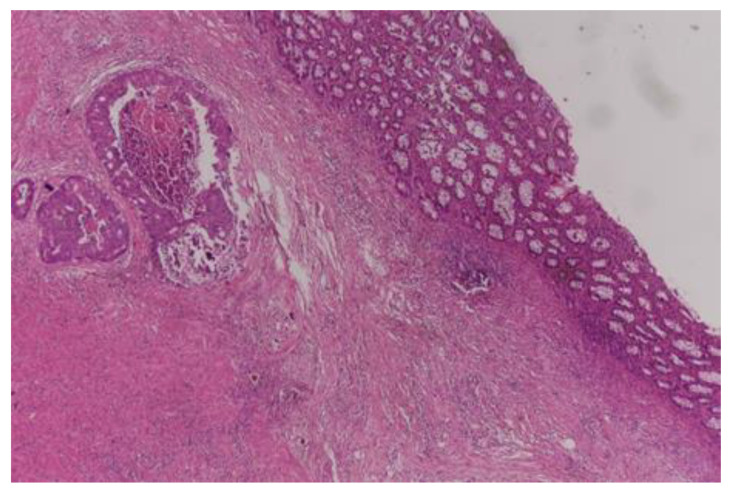
Minimal pathological response (TRG2); x 40 HE; Tumor in the submucosa, small foci of calcification.

**Figure 5:**
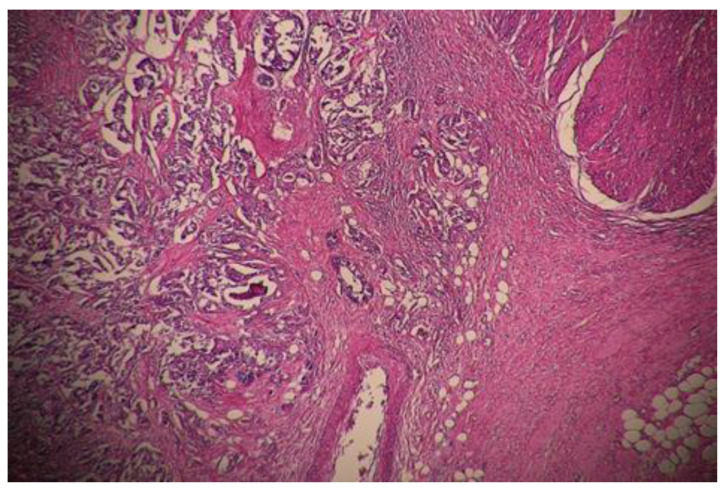
Poor pathological response (TRG3); x 40 HE; Widespread of residual cancer.

Complete pathological response (TRG0) was achieved in 29 (18.8%) patients (Figure 1), moderate response (TRG1) in 32 patients (Figure 2 and Figure 3), minimal response (TRG2) in 56 patients (Figure 4), and poor response (TRG3) in 37 patients (Figure 5). There was no statistical difference for the different pathologic response according to gender (*p* = 0.57), type of surgery (*p* = 0.29), and number of harvested lymph nodes (*p* = 0.93). More than half of the patients (20) with cPR presented with postoperative stage II and this difference in comparison to the rest represented statistical significance (*p* = 0.002).

The estimated survival according to the presence of LVI showed no statistical difference (*p* = 0.69, Mantel–Cox test). On the other hand, survival estimation with PNI presence was statistically significant (*p* = 0.001, Mantel–Cox test). The total mean survival for all the groups was 37.2 months. Survival within a different TRG score represented statistical significance (*p* = 0.006) (Table 2).

The multiple Cox proportional hazard regression analysis was used for multivariate analysis of factors affecting survival. It revealed that PNI (HR 2.042, 95% CI 1.031–4.046, *p* = 0.041) and tumor regression grade (HR 2.231, 95% CI 1.362–3.655, *p* = 0.001) significantly influence survival. Additionally, the overall multivariate model exhibited significance (Table 3).

**Figure 6:**
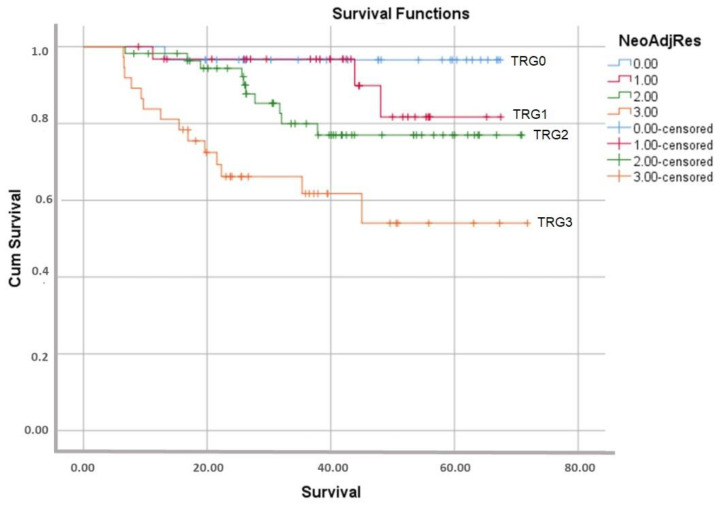
Kaplan–Meier curve on the overall survival between different TRGs.

The mean follow-up period was 41.7 months. The overall survival rate during the follow-up period was of 81.8%. The survival rates for patients with stage II and stage III cancer were of 89.2% and 76.4%, respectively. The estimated survival between the stages exhibited statistical significance according to the Mantel–Cox test (*p* = 0.04). The survival rates for different TRG scores were as follows: TRG0 (96.6%), TRG1 (90.6%), TRG2 (82.1%), and TRG3 (62.2%). The log-rank test presented this difference as highly statistically significant (*p* < 0.001), (Figure 6). The type of surgery did not affect the overall survival (*p* = 0.07).

## 4. Discussion

The concept of the cPR is clearly defined by the absence of residual tumor cells (ypT0N0M0) or by the presence of “in situ carcinoma” in the resected specimen with differences in the proposed classification systems [12]. A certain tumor regression in rectal cancer patients after the conduction of neoadjuvant CRT is expected. Patients with complete clinical response (cCR) can be considered for the “watch and wait” strategy in order to preserve the rectum and to not disturb the patients’ quality of life. This concept led to major changes in the approach of rectal cancer treatment. In the recent international multicenter register study from the International Watch & Wait Database (IWWD), the reported rate of cCR in 1009 patients treated with different neoadjuvant protocols was of 87.2%. However, the local tumor regrowth after cCR achievement is reported to be of 24.2% (mostly within two years after the treatment) and the distant metastasis occurrence rate was of 8% during follow-up [7].

Currently, there is no such diagnostic tool that can predict which patient will respond completely after the neoadjuvant CRT. Recently significant independent clinical predictors for cPR achievement were described, such as clinically negative lymph nodes, tumors smaller than 4 cm, and well differentiated tumors [24]. Other reported factors for complete pathological response prediction are free circumferential margin, absence of signet ring histology in specimen, tumor size, histology tumor, type and pretreatment clinical N stage [25,26].

The accent must be given to a factor that is not completely dependent on tumor biology and patient characteristics, i.e., the interval between neoadjuvant CRT conduction and surgery. In the meta-analysis of Petrelli et al., a surgery delay of more than 6–8 weeks after the completion of neoadjuvant CRT resulted in significant improvement of the cPR (*p* < 0.0001) with an increase in the rate from 13.7% to 19.5% [27]. Patients in this study were treated with surgery in the period of 8–12 weeks after neoadjuvant CRT completion.

However, prediction cannot be the basis for the treatment strategy on its own. Therefore, neoadjuvant chemoradiotherapy remains the standard treatment for locally advanced rectal cancer prior to surgery [18]. The type of neoadjuvant regimen is also still debatable. One dilemma is whether short-course (SC) radiotherapy or neoadjuvant long-course chemoradiotherapy will make a difference. A recent study by Wang et al. showed no differences in cPR rates and overall survival between two groups of patients with advanced rectal cancer receiving SC radiotherapy versus long-course CRT (*p*  =  0.510 and *p*  =  0.375, respectively) [28].

The second dilemma is which chemotherapy protocol is the best? The use of neoadjuvant FOLFOX protocol in combination with radiation therapy prior to radical rectal cancer surgery is shown to be feasible and safe with acceptable toxicity [29]. A recent study from Taiwan went as far as to include the FOLFOX regimen in the group of predictive factors for cPR achievement. Namely, their univariate analysis revealed the FOLFOX-based chemotherapy to be a favorable predictor of cPR (*p* = 0.041). The possible explanation of the authors from the mentioned study may be the biweekly use of FOLFOX during and after the radiation therapy [30]. FOLFOX was also used in this study.

No matter the proper use of different neoadjuvant regimens prior to radical rectal surgery, the problem of distant metastases development remains an unsolved issue. The inclusion of induction chemotherapy (trimodality treatment) as a new option against micrometastases was recently tested. Two studies failed to prove certain survival benefits [31,32]. The preliminary results of a phase II Italian study conducted on eight patients with the use of FOLFOXIRI (5-FU, leucovorin, oxaliplatin, and irinotecan) regimen in combination with targeted agents resulted in more than 50% tumor shrinkage, achievement of cCR in one patient, cPR in three patients, and nearly cPR in one patient [33].

Recent cPR rates after neoadjuvant CRT are reported to be between 10.4 and 33.9% [34,35,36,37,38,39,40]. The heterogeneity of the rates may be partially explained by the use of different TRG systems and the additional subgrouping of the patients with complete and near-complete/intermediate responses in one group [35,39,41]. This study presented a rate of cPR consistent with the current reports.

Different TRG systems were analyzed in terms of the number of categories included within them. It was suggested that the number heterogeneity of different TRG scores can lead to low concordance. The analysis of Trakarnsanga et al. concluded that the AJCC/CAP tumor regression grading (TRG) four-category system had the highest concordance index of 0.694 and was statistically significantly more accurate in predicting recurrence than the Mandard and the Dworak/Rödel systems [41]. Early reports with 2-year and 5-year follow-up periods showed improved overall survival, decrease in the local and distant recurrence rates, and high tumor-specific survival rates in patients with cPR [20,42,43]. Recent reports with follow-up periods up to 10 years are also in favor of the absence of local recurrence and an excellent disease-free survival rate of 95% [24,34,44]. This study also reports a high survival rate for patients with TRG0 and TRG1.

Further analyses in survival between cPR patients and those with partial response were conducted, thus showing differences in survival. Yang et al. grouped the TRGs 0, 1, and 2 and compared them with TRG 3 from the AJCC/CAP TRG system and performed a separate analysis for the pathologic stage ypII and ypIII of the resected specimen of rectal cancer patients. The univariate analysis presented a statistically significant association of the TRG with survival (*p* = 0.03). Similarly, in patients with ypIII, both univariate and multivariate analyses showed an association of the TRG with survival (*p* < 0.001) [35]. Sakin et al. used the Ryan (five categories) TRG criteria in their retrospective study on 182 patients. They found that the relapse-free survival and overall survival rates were significantly different among TRG groups (*p* = 0.003 and *p* = 0.008, respectively). In the mentioned study, different TRG scores were grouped into three categories (complete, intermediate, and poor response group) and were analyzed as such [36]. Palacios-Fuenmayor et al. achieved a rate of 17% for cPR. Their patients had higher disease-free survival in comparison to the others with partial or no response score [45]. By using the AJCC/CAP TRG system, in their multicenter cohort study, Chen et al. found statistically significant differences between all four categories of TRG and that the inferior ones were correlated with worse survival (disease-free survival, overall survival, local recurrence-free survival, and distant metastasis-free survival). In addition, the pairwise comparison of any two of the four categories had a distinguished outcome except for TRG1 vs. TRG2 for the local recurrence-free survival (*p* = 0.068) [15]. A similar type of TRG grouping into three categories conducted by Rödel was statistically significant after univariate analysis for disease-free survival (*p* = 0.006) and metastasis-free survival (*p* = 0.009) [38]. In the study by Li et al., patients were treated preoperatively with a two-week course of radiotherapy alone, thus achieving a cPR rate of 4.8%. It was shown that patients with complete/intermediate pathologic response had significantly improved overall survival, disease-free survival, and metastasis-free survival (*p* = 0.001, *p* < 0.001, and *p* < 0.001; respectively) [39]. Shin et al. identified preoperative CEA level, age ≥ 60, pCR, tumor differentiation, and PNI to be factors that affect the overall survival in their multivariate analysis [24]. In the present study, statistically significant differences in overall survival were proved among different TRG scores with the achievement of high survival rate for patients with TRG0 and TRG1 scores. At the same time, the multivariate analysis pointed to PNI and TRG as factors that affect survival.

This study contains limitations, namely this is a retrospective, single-center study. The number of patients is relatively small and the follow-up period is relatively short.

## 5. Conclusions

This study showed that the complete pathological response rate achievement after the use of long-course neoadjuvant chemoradiotherapy was of 18.8%. More than 50% of the patients with cPR exhibited postoperative stage II. Differences in TRG score had statistical significance in terms of overall survival. Higher TRG scores (TRG0 and TRG1) were associated with a survival rate of above 90% during follow-up and had better outcome, thus presenting as a significant prognostic factor in advanced rectal cancer patients treated with neoadjuvant long-course chemoradiotherapy. According to multivariate analysis, perineural invasion and tumor regression grade were found to be independent factors that affect survival in these patients.

Through the use of different chemotherapy regimens, induction chemotherapy, and the total neoadjuvant treatment, the response to these treatments is expected to result in higher rates of complete pathological and complete clinical response altogether. Consecutive studies will lead us to future follow-up and treatment strategies for the patients with advanced rectal cancer in order to achieve longer overall survival rates.

## Figures and Tables

**Table 1:** Patient, surgery, and tumor data.

	Total	Male	Female	*p* Value
Age, years (mean)	61.1	60.8	61.9	–
Type of surgery				
LAR ^1^	105	72	33	0.87 ^a^
APR/ELAPE ^2^	49	33	16	
Tumor stage				
II	65	46	19	0.55 ^a^
III	89	59	30	
Lymph node status				
N0	98	64	34	0.43 ^a^
N1, N2	56	40	16	
LVI ^3^ presence (%)	30 (19.5%)	22	8	0.48 ^a^
PNI ^4^ presence (%)	47 (30.5%)	35	12	0.32 ^a^

^1^ LAR = low anterior rectal resection; ^2^ APR/ELAPE = abdominoperineal rectal resection/extralevator abdominoperineal rectal resection; ^3^ LVI = lymphovascular invasion; ^4^ PNI = perineural invasion; ^a^ Pearson chi-squared test.

**Table 2:** Postoperative survival and surgery data according to TRG score.

	Tumor Regression Grade Score					
Variable	TRG0	TRG1	TRG2	TRG3	Total	*p* Value
Gender						
Male	18	23	41	23	105	0.57 ^a^
Female	11	9	15	14	49	
Total (%)	29 (18.8)	32 (20.7)	56 (36.3)	37 (24.2)		
Type of surgery						
LAR	24	20	36	25	105	0.29 ^a^
APR	5	12	20	12	49	
Neoadjuvant response according to stage						
Stage II	20	16	20	9	65	0.002 ^a^
Stage III	9	16	36	28	89	
Mean number of harvested lymph nodes	16.4	17.7	16.4	16.3	16.7	0.93 ^b^
Mean survival (months)	43.8	40.1	37.5	29.4	37.2	0.006 ^b^

TRG—tumor regression grade; ^a^ Pearson chi-squared test; ^b^ ANOVA test.

**Table 3:** Multivariate analysis of factors that affect survival.

Variables	Sig.	Hazard Ratio	95.0% CI ^1^ for Exp(B)		Omnibus Tests of Model Coefficients			
			Lower	Upper	−2 Log Likelihood	Chi-Squared	df	Sig.
LVI ^2^	0.542	0.740	0.281	1.946	263.394	21.871	5	0.001
PNI ^3^	0.041	2.042	1.031	4.046				
Stage	0.550	1.324	0.528	3.322				
MetLymph	0.940	0.996	0.907	1.095				
Pathologic response grade	0.001	2.231	1.362	3.655				

Multiple Cox proportional hazards regression analysis using the enter method, hazard ratio; ^1^ 95% CI = 95% confidence interval; ^2^ LVI = lymphovascular invasion; ^3^ PNI = perineural invasion.

## Data Availability

The data presented in this study are available on request from the corresponding author.
